# Observed data of extreme rainfall events over the West African Sahel

**DOI:** 10.1016/j.dib.2018.09.001

**Published:** 2018-09-06

**Authors:** Seyni Salack, Inoussa Abdou Saley, Jan Bliefernicht

**Affiliations:** a WASCAL Competence Center, Blvd Moammar El-khadafi, Ouagadougou 06, 06BP 9507 Ouagadougou, Burkina Faso; b Institute for Geography, University of Augsburg, Germany

**Keywords:** Percentile threshold, Extreme rainfall, West African Sahel

## Abstract

The data described in this article are sets of daily rainfall values derived from observed station records. The data was recorded by 72 in-situ rain gauges spread over the West African Sahel. The daily rainfall time series from synoptic, climate, agro-meteorological, and rainfall stations are assessed for quality and consistency before extreme values are extracted based on 90th, 95th, and 99th percentile thresholds. This data is free for use as part of the study "Scales for rating heavy rainfall events in West African Sahel" [1] (Salack et al., 2018). Complementary and up to date time series can be taken from WASCAL data infrastructure (WADI) geoportal https://wascal-dataportal.org/wascal_searchportal2/. This is a derived product (DP), made public in line with WASCAL׳s “3rd party data sharing policy” signed by the WASCAL member countries.

**Specifications table**

**Table:** 

Subject area	*Weather and climate extremes*
More specific subject area	*Rainfall extremes (disaster risk reduction)*
Type of data	*Tables (ASCII Tab-delimited text files)*
How data was acquired	*Statistically derived from daily records of raingauges*
Data format	*Raw measurement values (mm per day) of extreme daily rainfall events*
Experimental factors	*Daily time series of the rainfall amount from synoptic, climate, agro-meteorological and rainfall stations are assessed for quality and consistency. This was achieved through checking for erroneous measurement values dates and coordinates. A comprehensive visual inspection in combination with local meteorological expert knowledge and experience enabled the identification of outliers. Daily rainfall records were not interpolated when records are missing. Only the longest homogeneous time series, from 72 sites are processed.*
Experimental features	*The daily rainfall amounts are used to extract the corresponding 90th, 95th, and 99th percentiles threshold values.*
Data source location	*Sahel*
Data accessibility	*The rainfall extremes data are available with this article. Updated time series will be available taken from WASCAL data infrastructure (WADI) geoportal*https://wascal-dataportal.org/wascal_searchportal2/*or directly contacting individual meteorological services/agency of each member country.*
Related research article	Salack, S., Saley, A. I., Zabre, I., Zankli, L. N., Daaku, E. K. Scales for rating heavy rainfall events in the West African Sahel. Weather and Climate Extremes, 2018. https://doi.org/10.1016/j.wace.2018.05.004. [1]

**Value of the data**

• The data available with this article can be used in climate model diagnostics, output evaluation and hind-cast verification of forecasts.
• It can also serve as a benchmark information for an in-depth assessment of extreme rainfall events and can be used to support disaster risk reduction services delivery and the development of improved operational early warning services in the Sahel.
• The following activities can be supported by this dataset: Hydro-climatic diagnostics of extreme rainfall events. Climate models performance assessments. Hind-casts (forecasts) verification. Operational early warning service delivery. Event database and disaster risks reduction services planning and delivery.

## 1 Data

**Fig. 1:**
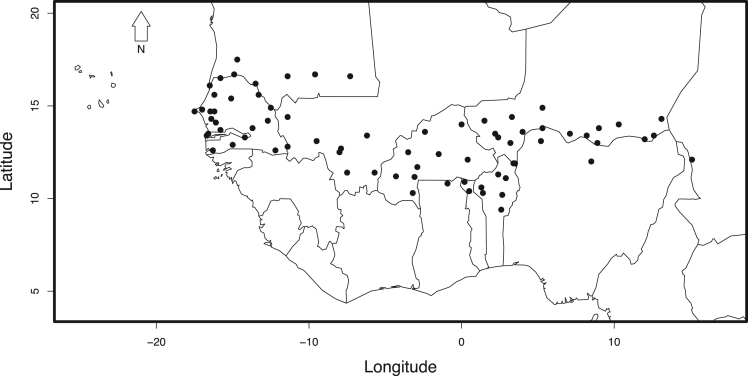
Spatial distribution of locations where different rain gauge types are recording extreme rainfall events in the West African Sahel.

The West African Sahel is defined as the sub-Saharan region that stretches from the western coasts of Senegal to the Central-Eastern edges of Chad between 10°N to 18°N. Observed daily rainfall records, of ordinary raingauges, weighing bucket and tipping bucket gauges*,* were provided by the meteorological services and agencies of the WASCAL member countries (www.wascal.org) following specific data sharing policies [1]. This database was complemented by daily measurements from the Global Historical Climatology Network [2] and the AMMA database (African Monsoon Multidisciplinary Analysis [3]). The daily rainfall time series (1960–2010) from synoptic, climate, agro-meteorological, and rainfall stations are assessed for quality and consistency before extreme values are extracted. This was achieved by checking erroneous measurement values (e.g., negative precipitation, temporal sequences with the same measurement value), dates and coordinates. The generation of multiple data plots enabled a comprehensive visual inspection of each time series. In combination with local meteorological knowledge and experience, the outliers, mostly caused by data entry typesetting errors, are identified and deleted [4]. Daily rainfall records are not interpolated when records are missing. Only the 72 longest time series, from different rain gauge types well-spread over the study area (Fig. 1), were selected to extract the datasets of daily rainfall extremes above the 90th, 95th, and 99th percentiles of each rainy season.

The data is stored as (1) *SahelRainfallR99P* (for the 99th percentile rainfall values), (2) *SahelRainfallR95P* (for the 95th percentile rainfall values) and (3) *SahelRainfallR90P* (for the 90th percentile rainfall values) with the following header:

– lon: Longitude
– lat: Latitude
– year: Year of data record
– doy: Day-of-year or Julian day of a calendar year (starting from 01-January)
– woy: Week-of-year or week number of a calendar year (starting from 01-January)
– int: Daily accumulated rainfall amount (mm/day)

The files are ASCII tab-delimited text (.txt) and missing values are tagged with “**NA**”

This is a derived product (DP), publicly available with this data article as part of the study presented by Salack et at. [1]. The data is in line with WASCAL׳s “3rd party data sharing policy” signed by the member countries of WASCAL during the establishment of new transboundary climate and hydro-meteorological observatories [1], [5]. Complementary and up to date time series for this dataset can be taken from the WASCAL data infrastructure (WADI) geoportal https://wascal-dataportal.org/wascal_searchportal2/.

## 2 Experimental design, materials, and method

**Fig. 2:**
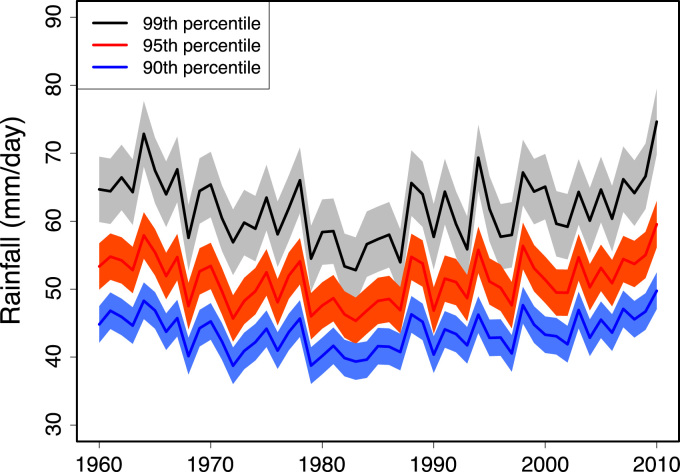
Inter-annual variability of average amounts of 99th, 95th, and 90th percentile threshold values defining extreme rainfall events over the West African Sahel. The shaded area is to the 95% confidence interval.

The scheme for the identification of extreme rainfall events was described by Salack et al. [1]. In this article a brief summary of this method is given. To extract precipitation measurements above the 99th (95th, 90th) percentile for each station, a vector of daily rainfall values RR (RR ≥ 1 mm) of each year was created and sorted in ascending order for each station. Then, we multiply 99% (95%, 90%) by the total number of those values of this vector to generate a rank index. The rank index is used to extract the corresponding value from the ordered vector based on the 99th (95th, 90th) percentile threshold value. The latter is used to extract all extreme rainfall events (ERE) greater or equal to it in each season׳s record. It is an improved peak-above-threshold method. Each ERE case(s) of a season is (are) identified with respect to the date(s)-of-occurrence (DTO) (DOY), the corresponding week(s)-of-the-year (WOY) and the daily amount(s) (INT). At each rain gauge location (longitude, latitude), any daily accumulated rainfall amount is considered as extreme rainfall if it belongs to the class of ERE which is greater than or equal to the 99th (95th, 90th) percentile (Fig. 2).
