# Supplementary material for: Observed data of extreme rainfall events over the West African Sahel
Source: Data Brief. 2018 Sep 6;20:1274–8. doi: 10.1016/j.dib.2018.09.001 (PMC6143746; doi:10.1016/j.dib.2018.09.001)
Supplement: Supplementary file 1 — Supplementary material [file mmc1.docx]

'Declarations of interest: none'.
