# Supplementary material for: Observed data of extreme rainfall events over the West African Sahel
Source: Data Brief. 2018 Sep 6;20:1274–8. doi: 10.1016/j.dib.2018.09.001 (PMC6143746; doi:10.1016/j.dib.2018.09.001)
Supplement: Supplementary file 2 — Supplementary material [file mmc2.zip › DIB-D-18-01349R2_Salacketal/Comments_DIB-D-18-01349R2.docx]

**DIB-D-18-01349**

**Title:** Observed daily amounts of extreme rainfall events over the West African Sahel

**Authors:** Seyni Salack, Inoussa Abdou Saley, Jan Bliefernicht

**Answers to comments**

Reviewers (if applicable):

Format Reviewer: Formatting edits required to comply with the Data in Brief format. The format reviewer has provided comments by editing the manuscript (Word with Track Changes). To view these comments: Go to folder "Submissions needing Revision". Expand the details, and click on "View review attachments". You can download the file provided by the format reviewer. See DIB-D-18-01349R1 format review.docx.

**>> The manuscript has been thoroughly revised and formatted according to format reviewer’s comments.**
