# Supplementary material for: Observed data of extreme rainfall events over the West African Sahel
Source: Data Brief. 2018 Sep 6;20:1274–8. doi: 10.1016/j.dib.2018.09.001 (PMC6143746; doi:10.1016/j.dib.2018.09.001)
Supplement: Supplementary file 2 — Supplementary material [file mmc2.zip › DIB-D-18-01349R2_Salacketal/Salacketal_DIB-D-18-01349R2.docx]

*Data article*

**Title: Observed data of extreme rainfall events over the West African Sahel**

**Authors:** Seyni Salack^a,*^, Inoussa Abdou Saley^a^ and Jan Bliefernicht^b^

**Affiliations:**

^a^WASCAL Competence Center, Blvd Moammar El-khadafi, 06BP 9507, Ouagadougou 06, Ouagadougou, Burkina Faso.

^b^ Institute for Geography, University of Augsburg, Germany.

**Contact email:** [salack.s@wascal.org](mailto:salack.s@wascal.org) / [abutawakalt@gmail.com](mailto:abutawakalt@gmail.com)

*Confidential manuscript submitted as “****Data in Brief****” to the journal of “****Weather and Climate Extremes****”*

**Abstract**

*The data described in this article are sets of daily rainfall values for the period 1960-2010. The data was recorded by 72 in-situ rain gauges spread over the West African Sahel. The daily rainfall time series from synoptic, climate, agro-meteorological, and rainfall stations are assessed for quality and consistency before extreme values are extracted based on 90^th^, 95^th^ and*

*99^th^ percentile thresholds. This data is free for use as part of the study “Scales for rating heavy rainfall events in the West African Sahel” [5]. Complementary and up to date time series can be taken from WASCAL data infrastructure (WADI) geoportal* [*https://wascal-dataportal.org/wascal_searchportal2/*](https://wascal-dataportal.org/wascal_searchportal2/)*. This is a derived product (DP), made public in line with WASCAL’s “3^rd^ party data sharing policy” signed by the WASCAL member countries.*

***Keywords****: Percentile Threshold, Extreme rainfall, West African Sahel*

**Specifications Table**

| Subject area | *Weather and climate extremes* |
| --- | --- |
| More specific subject area | *Rainfall extremes (disaster risk reduction)* |
| Type of data | *Tables (ASCII Tab-delimited text files)* |
| How data was acquired | *Statistically derived from daily records of raingauges* |
| Data format | *Raw measurement values (mm per day) of extreme daily rainfall events (1960-2010)* |
| Experimental factors | *Daily time series of the rainfall amount from synoptic, climate, agro-meteorological and rainfall stations are assessed for quality and consistency. This was achieved through checking for erroneous measurement values dates and coordinates. A comprehensive visual inspection in combination with local meteorological expert knowledge and experience enabled the identification of outliers. Daily rainfall records were not interpolated when records are missing. Only the longest homogeneous time series, from 72 sites are processed.* |
| Experimental features | *The daily rainfall amounts are used to extract the corresponding 99^th^, 95^th^and 90^th^ percentiles threshold values.* |
| Data source location | *Sahel* |
| Data accessibility | *The rainfall extremes data are available with this article. Updated time series will be available taken from WASCAL data infrastructure (WADI) geoportal* [*https://wascal-dataportal.org/wascal_searchportal2/*](https://wascal-dataportal.org/wascal_searchportal2/) *or directly contacting individual meteorological services/agency of each member country.* |
| Related research article | Salack, S., Saley, A. I., Zabre, I., Zankli, L. N., Daaku, E. K. Scales for rating heavy rainfall events in the West African Sahel. Weather and Climate Extremes, 2018. <https://doi.org/10.1016/j.wace.2018.05.004>. [1] |

**Value of the data**

The data available with this article can be used in climate model diagnostics, output evaluation and hind-cast verification of forecasts. It can also serve is a benchmark information for an in-depth assessment of extreme rainfall events and can be used to support disaster risk reduction services delivery and the development of improved operational early warning services in the Sahel. The following activities can be supported by this dataset:

- Hydro-climatic diagnostics of extreme rainfall events
- Climate models performance assessments
- Hind-casts (forecasts) verification
- Operational early warning service delivery
- Event database and disaster risks reduction services planning & delivery

**Data**

The West African Sahel is defined as the sub-Saharan region that stretches from the western coasts of Senegal to the Central-Eastern edges of Chad between 10°N to 18°N. Observed daily rainfall records, of ordinary raingauges, weighing bucket and tipping bucket gauges*,* were provided by the meteorological services and agencies of the WASCAL member countries ([www.wascal.org)](http://www.wascal.org)) following specific data sharing policies [1]. This database was complemented by daily measurements from the Global Historical Climatology Network[2] and the AMMA database (African Monsoon Multidisciplinary Analysis[3]). The daily rainfall time series (1960-2010) from synoptic, climate, agro-meteorological, and rainfall stations are assessed for quality and consistency before extreme values are extracted. This was achieved through checking erroneous measurement values (e.g. negative precipitation, temporal sequences with the same measurement value), dates and coordinates. The generation of multiple data plots enabled a comprehensive visual inspection of each time series. In combination with local meteorological knowledge and experience, the outliers, mostly caused by data entry typesetting errors, are identified and deleted[4]. Daily rainfall records are not interpolated when records are missing. Only the 72 longest time series, from different rain gauge types well-spread over the study area (Figure 1), were selected to extract the datasets of daily rainfall extremes above the 90^th^, 95^th^and 99^th^ percentiles of each rainy season.

The data is stored as (1) *SahelRainfallR99P* (for the 99^th^ percentile rainfall values), (2) *SahelRainfallR95P* (for the 95^th^ percentile rainfall values) and (3) *SahelRainfallR90P* (for the 90^th^ percentile rainfall values) with the following header:

- lon: Longitude
- lat: Latitude
- year: Year of data record
- doy: Day-of- -year or Julian day of a calendar year (starting from 01-January)
- woy: Week-of- -year or week number of a calendar year (starting from 01-January)
- int: Daily accumulated rainfall amount (mm/day)

The files are ASCII tab-delimited text (.txt) and missing values are tagged with “**NA**”

This is a derived product (DP), publicly available with this data article as part of the study presented by Salack et at.[1]. The data is in line with WASCAL’s “3^rd^ party data sharing policy” signed by the member countries of WASCAL during the establishment of new transboundary climate and hydro-meteorological observatories ([1], [5]). Complementary and up to date time series for this dataset can be taken from the WASCAL data infrastructure (WADI) geoportal <https://wascal-dataportal.org/wascal_searchportal2/>.

**

Figure 1: Spatial distribution of locations where different rain gauge types are recording extreme rainfall events in the West African Sahel.

**Experimental Design, Materials and Method**

The scheme for the identification of extreme rainfall events was described by Salack et al.[1]. In this article a brief summary of this method is given. To extract precipitation measurements above the 99^th^ (95^th^, 90^th^) percentile for each station, a vector of daily rainfall values RR (RR ≥ 1 mm) of each year was created and sorted in ascending order for each station. Then, we multiply 99% (95%, 90%) by the total number of those values of this vector to generate a rank index. The rank index is used to extract the corresponding value from the ordered vector based on the 99^th^ (95^th^, 90^th^) percentile threshold value. The latter is used to extract all extreme rainfall events (ERE) greater or equal to it in each season’s record. It is an improved peak-above-threshold method. Each ERE case(s) of a season is (are) identified with respect to the date(s)-of-occurrence (DTO) (DOY), the corresponding week(s)-of-the-year (WOY) and the daily amount(s) (INT). At each rain gauge location (longitude, latitude), any daily accumulated rainfall amount is considered as extreme rainfall if it belongs to the class of ERE which is greater than or equal to the 99^th^ (95^th^, 90^th^) percentile (figure 2).

Figure 2: Inter-annual variability of average amounts of 99^th^, 95^th^ & 90^th^ percentile threshold values defining extreme rainfall events over the West African Sahel. The shaded area is to the 95% confidence interval.

**Acknowledgements**

This work was partly supported by APTE-21/FSP-AGRICORA project funds (MEAE/IRD, AGRICORA axe 1, convention 2016-2018) provided by the French Ministry for Europe and Foreign Affairs (MEAE) through the *Institut de la Recherche pour le Développement* (<http://www.ird.fr/>).

The West African Science Center for Climate Change and Adapted Land Use ([www.wascal.org](http://www.wascal.org)) is sponsored by the German Federal Ministry of Education and Research (BMBF). We are thankful to all national meteorological services and agencies of West Africa for contributing the observed *in-situ* data.

**Declarations of interest: ‘**none**'**

**References**

1. Salack, S., Saley, A. I., Zabre, I., Zankli, L. N., Daaku, E. K. Scales for rating heavy rainfall events in the West African Sahel. Weather and Climate Extremes, 2018. <https://doi.org/10.1016/j.wace.2018.05.004>.
2. Menne, M. J., Durre, I., Vose, R. S., Gleason, B. E., and Houston, T. G. An overview of the global historical climatology network-daily database. *Journal of Atmospheric and Oceanic Technology*, 2012, 29 (7), 897-910. <https://doi.org/10.1175/JTECH-D-11-00103.1>
3. Fleury, L., et al. AMMA information system: an efficient cross-disciplinary tool and a legacy for forthcoming projects. *Atmosph. Sci. Lett.*, 2011, 12, 149–154. Doi:10.1002/asl.303.
4. Salack, S., Klein C., Giannini A., Sarr B., Worou O.N., Belko N., Bliefernicht J. and H. Kunstmann. Global warming induced hybrid rainy seasons in the Sahel, *Environ. Res. Lett.* 2016, 11, 10. <https://doi.org/10.1088/1748-9326/11/10/104008>
5. Bliefernicht, J., Berger, S., Salack, S., Guug, S., Hingerl L., Heinzeller, D., Mauder, M., Steinbrecher, R., Steub, G., Bossa, A., Waongo, M., Quansah, E., Balogun, A. A., Yira, Y., Arnault, J., Wagner, S., Klein, C., Straub, A., Schönrock, R., Kunkel, R., Rogmann, A., Neidl, F., Jahn, C., Diekkrüger, B., Aduna, A., Barry, B., Kunstmann, H. The WASCAL Hydro-Meteorological Observatory in the Sudan Savanna of Burkina Faso and Ghana, Vadoze Zone Journal. 2018 (Accepted).
